# Supplementary material for: HIV risk behavior and associated factors among people living with HIV/AIDS in Ethiopia: A systematic review and meta-analysis
Source: PLoS One. 2022 Jul 28;17(7):e0269304. doi: 10.1371/journal.pone.0269304 (PMC9333449; doi:10.1371/journal.pone.0269304)
Supplement: S1 File — (DOCX) [file pone.0269304.s002.docx]

Pub Med (3 articles)

*(((((((((((((("HIV risk behavior"[MeSH Terms])) OR ("high risk behavior"[MeSH Terms])) OR ("risky sexual practice"[MeSH Terms])) OR ("risky behavior"[MeSH Terms])) OR ("sexual behavior"[MeSH Terms])) OR ("unprotected sexual practice"[MeSH Terms])) OR ("condom use"[MeSH Terms])) OR ("inconsistent condom use"[MeSH Terms])) OR ("consistent condom use"[MeSH Terms])) OR ("casual sex"[MeSH Terms])) OR ("multiple sexual partner"[MeSH Terms])) AND ((((("alcohol use") OR ("alcohol consumption")) OR ("HIV status disclosure")) OR ("perceived stigma")) OR ("associated factors"))) AND ((((("HIV patients") OR ("AIDS patients")) OR ("people living with HIV/AIDS")) OR ("ART attendees")) OR ("HIV positive adults"))) AND ("Ethiopia")*

Filters applied: *English, Humans, from 2000/1/1 - 2020/9/30*.

Google scholar (37 articles)

**(("HIV risk behavior") OR ("high risk behavior") OR ("risky sexual practice") OR ("risky behavior") OR ("sexual behavior") OR ("unprotected sexual practice") OR ("condom use") OR ("inconsistent condom use") OR ("consistent condom use") OR ("casual sex") OR ("multiple sexual partner")) AND (("alcohol use") OR ("alcohol consumption") OR ("HIV status disclosure") OR ("perceived stigma") OR ("associated factors”)) AND (("HIV patients") OR ("AIDS patients") OR ("people living with HIV/AIDS") OR ("ART attendees") OR ("HIV positive adults")**

Filters applied: *Ethiopia, from 2000 - 2020*.

HINARI (3437 articles)

(("HIV risk behavior") OR ("high risk behavior") OR ("risky sexual practice") OR ("risky behavior") OR ("sexual behavior") OR ("unprotected sexual practice") OR ("condom use") OR ("inconsistent condom use") OR ("consistent condom use") OR ("casual sex") OR ("multiple sexual partner")) AND (("alcohol use") OR ("alcohol consumption") OR ("HIV status disclosure") OR ("perceived stigma") OR ("associated factors)) AND (("HIV positive patients") OR ("AIDS patients") OR ("people living with HIV/AIDS")) AND ("Ethiopia")

Filters applied: English, Humans, Medicine and Public Health,  *from 1/1/2000 - 9/30/2020*.

Cochrane Library (1 article)

**(("HIV risk behavior") OR ("high risk behavior") OR ("risky sexual practice") OR ("risky behavior") OR ("sexual behavior") OR ("unprotected sexual practice") OR ("condom use") OR ("inconsistent condom use") OR ("consistent condom use") OR ("casual sex") OR ("multiple sexual partner")) AND (("alcohol use") OR ("alcohol consumption") OR ("HIV status disclosure") OR ("perceived stigma") OR ("associated factors")) AND (("HIV patients") OR ("AIDS patients") OR ("people living with HIV/AIDS") OR ("ART attendees") OR ("HIV positive adults")) AND (“Ethiopia”)**

Filters applied: F*rom 2000 - 2020*.

CINAHL (293 articles)

(("HIV risk behavior") OR ("risky sexual practice") OR ("unprotected sexual practice") OR ("inconsistent condom use") OR ("consistent condom use")) AND (("alcohol use") AND ("HIV status disclosure") AND ("perceived stigma") OR ("associated factors")) AND (("HIV patients") OR ("people living with HIV/AIDS") AND (“Ethiopia”)

Filters applied: English, from *2000 - 2020*.

Global Health (366 articles)

("HIV risk behavior") OR ("high risk behavior") OR ("risky sexual practice") OR ("risky behavior") OR ("sexual behavior") OR ( "unprotected sexual practice") OR ( "condom use") OR ("inconsistent condom use") OR ( "consistent condom use",) OR ("casual sex") OR ("multiple sexual partner") AND ("alcohol use" ) AND ("HIV status disclosure") AND ( "alcohol consumption") AND ("perceived stigma") OR ("associated factors") AND ("HIV patients" ) OR ( "AIDS patients") OR ("people living with HIV/AIDS") OR ("ART attendees") OR ( "HIV positive adults") AND ("Ethiopia" ) AND yr:[2000 TO 2020]

Refined by: Language-English, geographic location-Ethiopia, publication year from *2000 - 2020*
