# Supplementary material for: HIV risk behavior and associated factors among people living with HIV/AIDS in Ethiopia: A systematic review and meta-analysis
Source: PLoS One. 2022 Jul 28;17(7):e0269304. doi: 10.1371/journal.pone.0269304 (PMC9333449; doi:10.1371/journal.pone.0269304)
Supplement: S2 File — (DOCX) [file pone.0269304.s003.docx]

**JBI Critical Appraisal Checklist for Prevalence Studies**

**Title: HIV risk behavior and its association with alcohol intake, HIV status non-disclosure, and perceived stigma among people living with HIV/AIDS in Ethiopia: A systematic review and meta-analysis.**

- The quality of each study was assessed by three authors (YD, BA, and MA) independently and 50% was used as a cut point for the inclusion of research articles. The differences in the result was settled by taking the mean score of the results of all reviewers

1. **Author: Dessie et al Year: 2011 Record Number: 1**

- Reviewer 1: Yitayish Damtie(YD) Date: 6/10/2020

|  |  | Yes | No | Unclear | N/A |
| --- | --- | --- | --- | --- | --- |
| 1 | Was the sample frame appropriate to address the target population? |  |  | X |  |
| 2 | Were study participants sampled in an appropriate way? | X |  |  |  |
| 3 | Was the sample size adequate? | X |  |  |  |
| 4 | Were the study subjects and the setting described in detail? | X |  |  |  |
| 5 | Was the data analysis conducted with sufficient coverage of the identified sample? | X |  |  |  |
| 6 | Were valid methods used for the identification of the condition? | X |  |  |  |
| 7 | Was  the  condition  measured  in  a  standard,  reliable  way   for  all  participants? | X |  |  |  |
| 8 | Was there appropriate statistical analysis? | X |  |  |  |
| 9 | Was the response rate adequate, and if not, was the low response rate managed appropriately? | X |  |  |  |

- Reviewer 2: Bezawit Adane(BA) Date: 8/10/2020

|  |  | Yes | No | Unclear | N/A |
| --- | --- | --- | --- | --- | --- |
| 1 | Was the sample frame appropriate to address the target population? |  |  | X |  |
| 2 | Were study participants sampled in an appropriate way? | X |  |  |  |
| 3 | Was the sample size adequate? | X |  |  |  |
| 4 | Were the study subjects and the setting described in detail? | X |  |  |  |
| 5 | Was the data analysis conducted with sufficient coverage of the identified sample? | X |  |  |  |
| 6 | Were valid methods used for the identification of the condition? |  |  | X |  |
| 7 | Was  the  condition  measured  in  a  standard,  reliable  way   for  all  participants? | X |  |  |  |
| 8 | Was there appropriate statistical analysis? | X |  |  |  |
| 9 | Was the response rate adequate, and if not, was the low response rate managed appropriately? | X |  |  |  |

- Reviewer 3: Mastewal Arefaynie(MA) Date: 7/10/2020

|  |  | Yes | No | Unclear | N/A |
| --- | --- | --- | --- | --- | --- |
| 1 | Was the sample frame appropriate to address the target population? |  |  | X |  |
| 2 | Were study participants sampled in an appropriate way? | X |  |  |  |
| 3 | Was the sample size adequate? | X |  |  |  |
| 4 | Were the study subjects and the setting described in detail? | X |  |  |  |
| 5 | Was the data analysis conducted with sufficient coverage of the identified sample? | X |  |  |  |
| 6 | Were valid methods used for the identification of the condition? | X |  |  |  |
| 7 | Was  the  condition  measured  in  a  standard,  reliable  way   for  all  participants? | X |  |  |  |
| 8 | Was there appropriate statistical analysis? | X |  |  |  |
| 9 | Was the response rate adequate, and if not, was the low response rate managed appropriately? | X |  |  |  |

**Overall appraisal: 85.2%, Include X Exclude □ Seek further info    □**

1. **Author: Tadesse and Gelagay Year: 2019 Record Number: 2**

- Reviewer 1: Yitayish Damtie(YD) Date: 6/10/2020

|  |  | Yes | No | Unclear | N/A |
| --- | --- | --- | --- | --- | --- |
| 1 | Was the sample frame appropriate to address the target population? |  |  | X |  |
| 2 | Were study participants sampled in an appropriate way? | X |  |  |  |
| 3 | Was the sample size adequate? | X |  |  |  |
| 4 | Were the study subjects and the setting described in detail? | X |  |  |  |
| 5 | Was the data analysis conducted with sufficient coverage of the identified sample? | X |  |  |  |
| 6 | Were valid methods used for the identification of the condition? | X |  |  |  |
| 7 | Was  the  condition  measured  in  a  standard,  reliable  way   for  all  participants? |  | X |  |  |
| 8 | Was there appropriate statistical analysis? | X |  |  |  |
| 9 | Was the response rate adequate, and if not, was the low response rate managed appropriately? | X |  |  |  |

- Reviewer 2: Bezawit Adane(BA) Date: 8/10/2020

|  |  | Yes | No | Unclear | N/A |
| --- | --- | --- | --- | --- | --- |
| 1 | Was the sample frame appropriate to address the target population? |  |  | X |  |
| 2 | Were study participants sampled in an appropriate way? | X |  |  |  |
| 3 | Was the sample size adequate? | X |  |  |  |
| 4 | Were the study subjects and the setting described in detail? | X |  |  |  |
| 5 | Was the data analysis conducted with sufficient coverage of the identified sample? | X |  |  |  |
| 6 | Were valid methods used for the identification of the condition? |  |  | X |  |
| 7 | Was  the  condition  measured  in  a  standard,  reliable  way   for  all  participants? | X |  |  |  |
| 8 | Was there appropriate statistical analysis? | X |  |  |  |
| 9 | Was the response rate adequate, and if not, was the low response rate managed appropriately? | X |  |  |  |

- Reviewer 3: Mastewal Arefaynie(MA) Date: 7/10/2020

|  |  | Yes | No | Unclear | N/A |
| --- | --- | --- | --- | --- | --- |
| 1 | Was the sample frame appropriate to address the target population? |  |  | X |  |
| 2 | Were study participants sampled in an appropriate way? | X |  |  |  |
| 3 | Was the sample size adequate? | X |  |  |  |
| 4 | Were the study subjects and the setting described in detail? | X |  |  |  |
| 5 | Was the data analysis conducted with sufficient coverage of the identified sample? | X |  |  |  |
| 6 | Were valid methods used for the identification of the condition? | X |  |  |  |
| 7 | Was  the  condition  measured  in  a  standard,  reliable  way   for  all  participants? | X |  |  |  |
| 8 | Was there appropriate statistical analysis? | X |  |  |  |
| 9 | Was the response rate adequate, and if not, was the low response rate managed appropriately? | X |  |  |  |

**Overall appraisal: 81.5%, Include X Exclude    □ Seek further info    □**

1. **Author: Alene et al Year: 2014 Record Number: 3**

- Reviewer 1: Yitayish Damtie(YD) Date: 6/10/2020

|  |  | Yes | No | Unclear | N/A |
| --- | --- | --- | --- | --- | --- |
| 1 | Was the sample frame appropriate to address the target population? |  |  | X |  |
| 2 | Were study participants sampled in an appropriate way? | X |  |  |  |
| 3 | Was the sample size adequate? |  | X |  |  |
| 4 | Were the study subjects and the setting described in detail? | X |  |  |  |
| 5 | Was the data analysis conducted with sufficient coverage of the identified sample? |  | X |  |  |
| 6 | Were valid methods used for the identification of the condition? | X |  |  |  |
| 7 | Was  the  condition  measured  in  a  standard,  reliable  way   for  all  participants? | X |  |  |  |
| 8 | Was there appropriate statistical analysis? | X |  |  |  |
| 9 | Was the response rate adequate, and if not, was the low response rate managed appropriately? |  | X |  |  |

- Reviewer 2: Bezawit Adane(BA) Date: 8/10/2020

|  |  | Yes | No | Unclear | N/A |
| --- | --- | --- | --- | --- | --- |
| 1 | Was the sample frame appropriate to address the target population? |  |  | X |  |
| 2 | Were study participants sampled in an appropriate way? | X |  |  |  |
| 3 | Was the sample size adequate? |  | X |  |  |
| 4 | Were the study subjects and the setting described in detail? | X |  |  |  |
| 5 | Was the data analysis conducted with sufficient coverage of the identified sample? | X |  |  |  |
| 6 | Were valid methods used for the identification of the condition? | X |  |  |  |
| 7 | Was  the  condition  measured  in  a  standard,  reliable  way   for  all  participants? | X |  |  |  |
| 8 | Was there appropriate statistical analysis? | X |  |  |  |
| 9 | Was the response rate adequate, and if not, was the low response rate managed appropriately? |  | X |  |  |

- Reviewer 3: Mastewal Arefaynie(MA) Date: 7/10/2020

|  |  | Yes | No | Unclear | N/A |
| --- | --- | --- | --- | --- | --- |
| 1 | Was the sample frame appropriate to address the target population? |  |  | X |  |
| 2 | Were study participants sampled in an appropriate way? | X |  |  |  |
| 3 | Was the sample size adequate? |  | X |  |  |
| 4 | Were the study subjects and the setting described in detail? | X |  |  |  |
| 5 | Was the data analysis conducted with sufficient coverage of the identified sample? | X |  |  |  |
| 6 | Were valid methods used for the identification of the condition? | X |  |  |  |
| 7 | Was  the  condition  measured  in  a  standard,  reliable  way   for  all  participants? | X |  |  |  |
| 8 | Was there appropriate statistical analysis? | X |  |  |  |
| 9 | Was the response rate adequate, and if not, was the low response rate managed appropriately? |  | X |  |  |

**Overall appraisal: 63%, Include X Exclude    □ Seek further info    □**

1. **Author: Engedashet et al Year: 2014 Record Number: 4**

- Reviewer 1: Yitayish Damtie(YD) Date: 6/10/2020

|  |  | Yes | No | Unclear | N/A |
| --- | --- | --- | --- | --- | --- |
| 1 | Was the sample frame appropriate to address the target population? |  |  | X |  |
| 2 | Were study participants sampled in an appropriate way? | X |  |  |  |
| 3 | Was the sample size adequate? | X |  |  |  |
| 4 | Were the study subjects and the setting described in detail? | X |  |  |  |
| 5 | Was the data analysis conducted with sufficient coverage of the identified sample? | X |  |  |  |
| 6 | Were valid methods used for the identification of the condition? | X |  |  |  |
| 7 | Was  the  condition  measured  in  a  standard,  reliable  way   for  all  participants? | X |  |  |  |
| 8 | Was there appropriate statistical analysis? | X |  |  |  |
| 9 | Was the response rate adequate, and if not, was the low response rate managed appropriately? | X |  |  |  |

- Reviewer 2: Bezawit Adane(BA) Date: 8/10/2020

|  |  | Yes | No | Unclear | N/A |
| --- | --- | --- | --- | --- | --- |
| 1 | Was the sample frame appropriate to address the target population? |  |  | X |  |
| 2 | Were study participants sampled in an appropriate way? | X |  |  |  |
| 3 | Was the sample size adequate? | X |  |  |  |
| 4 | Were the study subjects and the setting described in detail? | X |  |  |  |
| 5 | Was the data analysis conducted with sufficient coverage of the identified sample? | X |  |  |  |
| 6 | Were valid methods used for the identification of the condition? |  |  | X |  |
| 7 | Was  the  condition  measured  in  a  standard,  reliable  way   for  all  participants? | X |  |  |  |
| 8 | Was there appropriate statistical analysis? | X |  |  |  |
| 9 | Was the response rate adequate, and if not, was the low response rate managed appropriately? | X |  |  |  |

- Reviewer 3: Mastewal Arefaynie(MA) Date: 7/10/2020

|  |  | Yes | No | Unclear | N/A |
| --- | --- | --- | --- | --- | --- |
| 1 | Was the sample frame appropriate to address the target population? |  |  | X |  |
| 2 | Were study participants sampled in an appropriate way? | X |  |  |  |
| 3 | Was the sample size adequate? | X |  |  |  |
| 4 | Were the study subjects and the setting described in detail? | X |  |  |  |
| 5 | Was the data analysis conducted with sufficient coverage of the identified sample? | X |  |  |  |
| 6 | Were valid methods used for the identification of the condition? | X |  |  |  |
| 7 | Was  the  condition  measured  in  a  standard,  reliable  way   for  all  participants? | X |  |  |  |
| 8 | Was there appropriate statistical analysis? | X |  |  |  |
| 9 | Was the response rate adequate, and if not, was the low response rate managed appropriately? | X |  |  |  |

**Overall appraisal: 85.2%,   Include X Exclude    □ Seek further info    □**

1. **Author: Demissie et al Year: 2015 Record Number: 5**

- Reviewer 1: Yitayish Damtie(YD) Date: 6/10/2020

|  |  | Yes | No | Unclear | N/A |
| --- | --- | --- | --- | --- | --- |
| 1 | Was the sample frame appropriate to address the target population? |  |  | X |  |
| 2 | Were study participants sampled in an appropriate way? | X |  |  |  |
| 3 | Was the sample size adequate? |  | X |  |  |
| 4 | Were the study subjects and the setting described in detail? | X |  |  |  |
| 5 | Was the data analysis conducted with sufficient coverage of the identified sample? |  | X |  |  |
| 6 | Were valid methods used for the identification of the condition? | X |  |  |  |
| 7 | Was  the  condition  measured  in  a  standard,  reliable  way   for  all  participants? | X |  |  |  |
| 8 | Was there appropriate statistical analysis? | X |  |  |  |
| 9 | Was the response rate adequate, and if not, was the low response rate managed appropriately? | X |  |  |  |

- Reviewer 2: Bezawit Adane(BA) Date: 8/10/2020

|  |  | Yes | No | Unclear | N/A |
| --- | --- | --- | --- | --- | --- |
| 1 | Was the sample frame appropriate to address the target population? |  |  | X |  |
| 2 | Were study participants sampled in an appropriate way? | X |  |  |  |
| 3 | Was the sample size adequate? |  | X |  |  |
| 4 | Were the study subjects and the setting described in detail? | X |  |  |  |
| 5 | Was the data analysis conducted with sufficient coverage of the identified sample? |  | X |  |  |
| 6 | Were valid methods used for the identification of the condition? | X |  |  |  |
| 7 | Was  the  condition  measured  in  a  standard,  reliable  way   for  all  participants? | X |  |  |  |
| 8 | Was there appropriate statistical analysis? | X |  |  |  |
| 9 | Was the response rate adequate, and if not, was the low response rate managed appropriately? | X |  |  |  |

- Reviewer 3: Mastewal Arefaynie(MA) Date: 7/10/2020

|  |  | Yes | No | Unclear | N/A |
| --- | --- | --- | --- | --- | --- |
| 1 | Was the sample frame appropriate to address the target population? |  |  | X |  |
| 2 | Were study participants sampled in an appropriate way? | X |  |  |  |
| 3 | Was the sample size adequate? |  | X |  |  |
| 4 | Were the study subjects and the setting described in detail? | X |  |  |  |
| 5 | Was the data analysis conducted with sufficient coverage of the identified sample? |  | X |  |  |
| 6 | Were valid methods used for the identification of the condition? | X |  |  |  |
| 7 | Was  the  condition  measured  in  a  standard,  reliable  way   for  all  participants? | X |  |  |  |
| 8 | Was there appropriate statistical analysis? | X |  |  |  |
| 9 | Was the response rate adequate, and if not, was the low response rate managed appropriately? | X |  |  |  |

**Overall appraisal: 66.7%, Include X Exclude    □ Seek further info    □**

1. **Author: Molla and Gelagay Year: 2017 Record Number: 6**

- Reviewer 1: Yitayish Damtie(YD) Date: 6/10/2020

|  |  | Yes | No | Unclear | N/A |
| --- | --- | --- | --- | --- | --- |
| 1 | Was the sample frame appropriate to address the target population? |  |  | X |  |
| 2 | Were study participants sampled in an appropriate way? | X |  |  |  |
| 3 | Was the sample size adequate? | X |  |  |  |
| 4 | Were the study subjects and the setting described in detail? | X |  |  |  |
| 5 | Was the data analysis conducted with sufficient coverage of the identified sample? | X |  |  |  |
| 6 | Were valid methods used for the identification of the condition? | X |  |  |  |
| 7 | Was  the  condition  measured  in  a  standard,  reliable  way   for  all  participants? |  | X |  |  |
| 8 | Was there appropriate statistical analysis? | X |  |  |  |
| 9 | Was the response rate adequate, and if not, was the low response rate managed appropriately? | X |  |  |  |

- Reviewer 2: Bezawit Adane(BA) Date: 8/10/2020

|  |  | Yes | No | Unclear | N/A |
| --- | --- | --- | --- | --- | --- |
| 1 | Was the sample frame appropriate to address the target population? |  |  | X |  |
| 2 | Were study participants sampled in an appropriate way? | X |  |  |  |
| 3 | Was the sample size adequate? |  | X |  |  |
| 4 | Were the study subjects and the setting described in detail? | X |  |  |  |
| 5 | Was the data analysis conducted with sufficient coverage of the identified sample? | X |  |  |  |
| 6 | Were valid methods used for the identification of the condition? |  |  | X |  |
| 7 | Was  the  condition  measured  in  a  standard,  reliable  way   for  all  participants? | X |  |  |  |
| 8 | Was there appropriate statistical analysis? | X |  |  |  |
| 9 | Was the response rate adequate, and if not, was the low response rate managed appropriately? | X |  |  |  |

- Reviewer 3: Mastewal Arefaynie(MA) Date: 7/10/2020

|  |  | Yes | No | Unclear | N/A |
| --- | --- | --- | --- | --- | --- |
| 1 | Was the sample frame appropriate to address the target population? |  |  | X |  |
| 2 | Were study participants sampled in an appropriate way? | X |  |  |  |
| 3 | Was the sample size adequate? | X |  |  |  |
| 4 | Were the study subjects and the setting described in detail? | X |  |  |  |
| 5 | Was the data analysis conducted with sufficient coverage of the identified sample? | X |  |  |  |
| 6 | Were valid methods used for the identification of the condition? | X |  |  |  |
| 7 | Was  the  condition  measured  in  a  standard,  reliable  way   for  all  participants? | X |  |  |  |
| 8 | Was there appropriate statistical analysis? | X |  |  |  |
| 9 | Was the response rate adequate, and if not, was the low response rate managed appropriately? | X |  |  |  |

**Overall appraisal: 77 .8%, Include X Exclude    □ Seek further info    □**

1. **Author: Mosisa et al Year: 2018 Record Number: 7**

- Reviewer 1: Yitayish Damtie(YD) Date: 6/10/2020

|  |  | Yes | No | Unclear | N/A |
| --- | --- | --- | --- | --- | --- |
| 1 | Was the sample frame appropriate to address the target population? |  |  | X |  |
| 2 | Were study participants sampled in an appropriate way? | X |  |  |  |
| 3 | Was the sample size adequate? |  | X |  |  |
| 4 | Were the study subjects and the setting described in detail? | X |  |  |  |
| 5 | Was the data analysis conducted with sufficient coverage of the identified sample? |  | X |  |  |
| 6 | Were valid methods used for the identification of the condition? | X |  |  |  |
| 7 | Was  the  condition  measured  in  a  standard,  reliable  way   for  all  participants? | X |  |  |  |
| 8 | Was there appropriate statistical analysis? | X |  |  |  |
| 9 | Was the response rate adequate, and if not, was the low response rate managed appropriately? | X |  |  |  |

- Reviewer 2: Bezawit Adane(BA) Date: 8/10/2020

|  |  | Yes | No | Unclear | N/A |
| --- | --- | --- | --- | --- | --- |
| 1 | Was the sample frame appropriate to address the target population? |  |  | X |  |
| 2 | Were study participants sampled in an appropriate way? | X |  |  |  |
| 3 | Was the sample size adequate? |  | X |  |  |
| 4 | Were the study subjects and the setting described in detail? | X |  |  |  |
| 5 | Was the data analysis conducted with sufficient coverage of the identified sample? |  | X |  |  |
| 6 | Were valid methods used for the identification of the condition? | X |  |  |  |
| 7 | Was  the  condition  measured  in  a  standard,  reliable  way   for  all  participants? | X |  |  |  |
| 8 | Was there appropriate statistical analysis? | X |  |  |  |
| 9 | Was the response rate adequate, and if not, was the low response rate managed appropriately? | X |  |  |  |

- Reviewer 3: Mastewal Arefaynie(MA) Date: 7/10/2020

|  |  | Yes | No | Unclear | N/A |
| --- | --- | --- | --- | --- | --- |
| 1 | Was the sample frame appropriate to address the target population? |  |  | X |  |
| 2 | Were study participants sampled in an appropriate way? | X |  |  |  |
| 3 | Was the sample size adequate? |  | X |  |  |
| 4 | Were the study subjects and the setting described in detail? | X |  |  |  |
| 5 | Was the data analysis conducted with sufficient coverage of the identified sample? |  | X |  |  |
| 6 | Were valid methods used for the identification of the condition? | X |  |  |  |
| 7 | Was  the  condition  measured  in  a  standard,  reliable  way   for  all  participants? | X |  |  |  |
| 8 | Was there appropriate statistical analysis? | X |  |  |  |
| 9 | Was the response rate adequate, and if not, was the low response rate managed appropriately? | X |  |  |  |

**Overall appraisal: 66.7%, Include X Exclude    □ Seek further info    □**

1. **Author: Ali et al Year: 2019 Record Number: 8**

- Reviewer 1: Yitayish Damtie(YD) Date: 6/10/2020

|  |  | Yes | No | Unclear | N/A |
| --- | --- | --- | --- | --- | --- |
| 1 | Was the sample frame appropriate to address the target population? |  |  | X |  |
| 2 | Were study participants sampled in an appropriate way? | X |  |  |  |
| 3 | Was the sample size adequate? |  | X |  |  |
| 4 | Were the study subjects and the setting described in detail? | X |  |  |  |
| 5 | Was the data analysis conducted with sufficient coverage of the identified sample? |  | X |  |  |
| 6 | Were valid methods used for the identification of the condition? | X |  |  |  |
| 7 | Was  the  condition  measured  in  a  standard,  reliable  way   for  all  participants? | X |  |  |  |
| 8 | Was there appropriate statistical analysis? | X |  |  |  |
| 9 | Was the response rate adequate, and if not, was the low response rate managed appropriately? |  | X |  |  |

- Reviewer 2: Bezawit Adane(BA) Date: 8/10/2020

|  |  | Yes | No | Unclear | N/A |
| --- | --- | --- | --- | --- | --- |
| 1 | Was the sample frame appropriate to address the target population? |  |  | X |  |
| 2 | Were study participants sampled in an appropriate way? | X |  |  |  |
| 3 | Was the sample size adequate? |  | X |  |  |
| 4 | Were the study subjects and the setting described in detail? | X |  |  |  |
| 5 | Was the data analysis conducted with sufficient coverage of the identified sample? |  | X |  |  |
| 6 | Were valid methods used for the identification of the condition? | X |  |  |  |
| 7 | Was  the  condition  measured  in  a  standard,  reliable  way   for  all  participants? | X |  |  |  |
| 8 | Was there appropriate statistical analysis? | X |  |  |  |
| 9 | Was the response rate adequate, and if not, was the low response rate managed appropriately? |  | X |  |  |

- Reviewer 3: Mastewal Arefaynie(MA) Date: 7/10/2020

|  |  | Yes | No | Unclear | N/A |
| --- | --- | --- | --- | --- | --- |
| 1 | Was the sample frame appropriate to address the target population? | X |  |  |  |
| 2 | Were study participants sampled in an appropriate way? | X |  |  |  |
| 3 | Was the sample size adequate? |  | X |  |  |
| 4 | Were the study subjects and the setting described in detail? | X |  |  |  |
| 5 | Was the data analysis conducted with sufficient coverage of the identified sample? |  | X |  |  |
| 6 | Were valid methods used for the identification of the condition? | X |  |  |  |
| 7 | Was  the  condition  measured  in  a  standard,  reliable  way   for  all  participants? | X |  |  |  |
| 8 | Was there appropriate statistical analysis? | X |  |  |  |
| 9 | Was the response rate adequate, and if not, was the low response rate managed appropriately? |  | X |  |  |

**Overall appraisal: 59.2%, Include X Exclude    □ Seek further info    □**

1. **Author: Shewamene et al Year: 2015 Record Number: 9**

- Reviewer 1: Yitayish Damtie(YD) Date: 6/10/2020

|  |  | Yes | No | Unclear | N/A |
| --- | --- | --- | --- | --- | --- |
| 1 | Was the sample frame appropriate to address the target population? |  |  | X |  |
| 2 | Were study participants sampled in an appropriate way? | X |  |  |  |
| 3 | Was the sample size adequate? |  | X |  |  |
| 4 | Were the study subjects and the setting described in detail? | X |  |  |  |
| 5 | Was the data analysis conducted with sufficient coverage of the identified sample? |  | X |  |  |
| 6 | Were valid methods used for the identification of the condition? | X |  |  |  |
| 7 | Was  the  condition  measured  in  a  standard,  reliable  way   for  all  participants? | X |  |  |  |
| 8 | Was there appropriate statistical analysis? | X |  |  |  |
| 9 | Was the response rate adequate, and if not, was the low response rate managed appropriately? | X |  |  |  |

- Reviewer 2: Bezawit Adane(BA) Date: 8/10/2020

|  |  | Yes | No | Unclear | N/A |
| --- | --- | --- | --- | --- | --- |
| 1 | Was the sample frame appropriate to address the target population? |  |  | X |  |
| 2 | Were study participants sampled in an appropriate way? | X |  |  |  |
| 3 | Was the sample size adequate? |  | X |  |  |
| 4 | Were the study subjects and the setting described in detail? | X |  |  |  |
| 5 | Was the data analysis conducted with sufficient coverage of the identified sample? |  | X |  |  |
| 6 | Were valid methods used for the identification of the condition? | X |  |  |  |
| 7 | Was  the  condition  measured  in  a  standard,  reliable  way   for  all  participants? | X |  |  |  |
| 8 | Was there appropriate statistical analysis? | X |  |  |  |
| 9 | Was the response rate adequate, and if not, was the low response rate managed appropriately? | X |  |  |  |

- Reviewer 3: Mastewal Arefaynie(MA) Date: 7/10/2020

|  |  | Yes | No | Unclear | N/A |
| --- | --- | --- | --- | --- | --- |
| 1 | Was the sample frame appropriate to address the target population? |  |  | X |  |
| 2 | Were study participants sampled in an appropriate way? | X |  |  |  |
| 3 | Was the sample size adequate? |  | X |  |  |
| 4 | Were the study subjects and the setting described in detail? | X |  |  |  |
| 5 | Was the data analysis conducted with sufficient coverage of the identified sample? |  | X |  |  |
| 6 | Were valid methods used for the identification of the condition? | X |  |  |  |
| 7 | Was  the  condition  measured  in  a  standard,  reliable  way   for  all  participants? | X |  |  |  |
| 8 | Was there appropriate statistical analysis? | X |  |  |  |
| 9 | Was the response rate adequate, and if not, was the low response rate managed appropriately? | X |  |  |  |

**Overall appraisal: 66.7%, Include X Exclude    □ Seek further info    □**

1. **Author: Yalew et al Year: 2012 Record Number: 10**

- Reviewer 1: Yitayish Damtie(YD) Date: 6/10/2020

|  |  | Yes | No | Unclear | N/A |
| --- | --- | --- | --- | --- | --- |
| 1 | Was the sample frame appropriate to address the target population? |  |  | X |  |
| 2 | Were study participants sampled in an appropriate way? | X |  |  |  |
| 3 | Was the sample size adequate? |  | X |  |  |
| 4 | Were the study subjects and the setting described in detail? | X |  |  |  |
| 5 | Was the data analysis conducted with sufficient coverage of the identified sample? |  | X |  |  |
| 6 | Were valid methods used for the identification of the condition? |  | X |  |  |
| 7 | Was  the  condition  measured  in  a  standard,  reliable  way   for  all  participants? | X |  |  |  |
| 8 | Was there appropriate statistical analysis? | X |  |  |  |
| 9 | Was the response rate adequate, and if not, was the low response rate managed appropriately? | X |  |  |  |

- Reviewer 2: Bezawit Adane(BA) Date: 8/10/2020

|  |  | Yes | No | Unclear | N/A |
| --- | --- | --- | --- | --- | --- |
| 1 | Was the sample frame appropriate to address the target population? |  |  | X |  |
| 2 | Were study participants sampled in an appropriate way? | X |  |  |  |
| 3 | Was the sample size adequate? |  | X |  |  |
| 4 | Were the study subjects and the setting described in detail? | X |  |  |  |
| 5 | Was the data analysis conducted with sufficient coverage of the identified sample? |  | X |  |  |
| 6 | Were valid methods used for the identification of the condition? |  | X |  |  |
| 7 | Was  the  condition  measured  in  a  standard,  reliable  way   for  all  participants? | X |  |  |  |
| 8 | Was there appropriate statistical analysis? | X |  |  |  |
| 9 | Was the response rate adequate, and if not, was the low response rate managed appropriately? | X |  |  |  |

- Reviewer 3: Mastewal Arefaynie(MA) Date: 7/10/2020

|  |  | Yes | No | Unclear | N/A |
| --- | --- | --- | --- | --- | --- |
| 1 | Was the sample frame appropriate to address the target population? |  | X |  |  |
| 2 | Were study participants sampled in an appropriate way? | X |  |  |  |
| 3 | Was the sample size adequate? |  | X |  |  |
| 4 | Were the study subjects and the setting described in detail? | X |  |  |  |
| 5 | Was the data analysis conducted with sufficient coverage of the identified sample? |  | X |  |  |
| 6 | Were valid methods used for the identification of the condition? | X |  |  |  |
| 7 | Was  the  condition  measured  in  a  standard,  reliable  way   for  all  participants? |  | X |  |  |
| 8 | Was there appropriate statistical analysis? | X |  |  |  |
| 9 | Was the response rate adequate, and if not, was the low response rate managed appropriately? | X |  |  |  |

**Overall appraisal: 55.6%, Include X Exclude    □ Seek further info    □**

1. **Author: Yalew et al Year: 2012 Record Number: 11**

- Reviewer 1: Yitayish Damtie(YD) Date: 6/10/2020

|  |  | Yes | No | Unclear | N/A |
| --- | --- | --- | --- | --- | --- |
| 1 | Was the sample frame appropriate to address the target population? |  |  | X |  |
| 2 | Were study participants sampled in an appropriate way? | X |  |  |  |
| 3 | Was the sample size adequate? |  | X |  |  |
| 4 | Were the study subjects and the setting described in detail? | X |  |  |  |
| 5 | Was the data analysis conducted with sufficient coverage of the identified sample? |  | X |  |  |
| 6 | Were valid methods used for the identification of the condition? |  | X |  |  |
| 7 | Was  the  condition  measured  in  a  standard,  reliable  way   for  all  participants? | X |  |  |  |
| 8 | Was there appropriate statistical analysis? | X |  |  |  |
| 9 | Was the response rate adequate, and if not, was the low response rate managed appropriately? | X |  |  |  |

- Reviewer 2: Bezawit Adane(BA) Date: 8/10/2020

|  |  | Yes | No | Unclear | N/A |
| --- | --- | --- | --- | --- | --- |
| 1 | Was the sample frame appropriate to address the target population? |  |  | X |  |
| 2 | Were study participants sampled in an appropriate way? | X |  |  |  |
| 3 | Was the sample size adequate? |  | X |  |  |
| 4 | Were the study subjects and the setting described in detail? | X |  |  |  |
| 5 | Was the data analysis conducted with sufficient coverage of the identified sample? |  | X |  |  |
| 6 | Were valid methods used for the identification of the condition? |  | X |  |  |
| 7 | Was  the  condition  measured  in  a  standard,  reliable  way   for  all  participants? | X |  |  |  |
| 8 | Was there appropriate statistical analysis? | X |  |  |  |
| 9 | Was the response rate adequate, and if not, was the low response rate managed appropriately? | X |  |  |  |

- Reviewer 3: Mastewal Arefaynie(MA) Date: 7/10/2020

|  |  | Yes | No | Unclear | N/A |
| --- | --- | --- | --- | --- | --- |
| 1 | Was the sample frame appropriate to address the target population? |  | X |  |  |
| 2 | Were study participants sampled in an appropriate way? | X |  |  |  |
| 3 | Was the sample size adequate? |  | X |  |  |
| 4 | Were the study subjects and the setting described in detail? | X |  |  |  |
| 5 | Was the data analysis conducted with sufficient coverage of the identified sample? |  | X |  |  |
| 6 | Were valid methods used for the identification of the condition? | X |  |  |  |
| 7 | Was  the  condition  measured  in  a  standard,  reliable  way   for  all  participants? |  | X |  |  |
| 8 | Was there appropriate statistical analysis? | X |  |  |  |
| 9 | Was the response rate adequate, and if not, was the low response rate managed appropriately? | X |  |  |  |

**Overall appraisal: 55.6%, Include X Exclude    □ Seek further info    □**

1. **Author: Deribe et al Year: 2008 Record Number: 12**

- Reviewer 1: Yitayish Damtie(YD) Date: 6/10/2020

|  |  | Yes | No | Unclear | N/A |
| --- | --- | --- | --- | --- | --- |
| 1 | Was the sample frame appropriate to address the target population? |  |  | X |  |
| 2 | Were study participants sampled in an appropriate way? | X |  |  |  |
| 3 | Was the sample size adequate? | X |  |  |  |
| 4 | Were the study subjects and the setting described in detail? | X |  |  |  |
| 5 | Was the data analysis conducted with sufficient coverage of the identified sample? | X |  |  |  |
| 6 | Were valid methods used for the identification of the condition? | X |  |  |  |
| 7 | Was  the  condition  measured  in  a  standard,  reliable  way   for  all  participants? | X |  |  |  |
| 8 | Was there appropriate statistical analysis? | X |  |  |  |
| 9 | Was the response rate adequate, and if not, was the low response rate managed appropriately? | X |  |  |  |

- Reviewer 2: Bezawit Adane(BA) Date: 8/10/2020

|  |  | Yes | No | Unclear | N/A |
| --- | --- | --- | --- | --- | --- |
| 1 | Was the sample frame appropriate to address the target population? |  |  | X |  |
| 2 | Were study participants sampled in an appropriate way? | X |  |  |  |
| 3 | Was the sample size adequate? | X |  |  |  |
| 4 | Were the study subjects and the setting described in detail? | X |  |  |  |
| 5 | Was the data analysis conducted with sufficient coverage of the identified sample? | X |  |  |  |
| 6 | Were valid methods used for the identification of the condition? | x |  |  |  |
| 7 | Was  the  condition  measured  in  a  standard,  reliable  way   for  all  participants? | X |  |  |  |
| 8 | Was there appropriate statistical analysis? | X |  |  |  |
| 9 | Was the response rate adequate, and if not, was the low response rate managed appropriately? | X |  |  |  |

- Reviewer 3: Mastewal Arefaynie(MA) Date: 7/10/2020

|  |  | Yes | No | Unclear | N/A |
| --- | --- | --- | --- | --- | --- |
| 1 | Was the sample frame appropriate to address the target population? |  |  | X |  |
| 2 | Were study participants sampled in an appropriate way? | X |  |  |  |
| 3 | Was the sample size adequate? | X |  |  |  |
| 4 | Were the study subjects and the setting described in detail? | X |  |  |  |
| 5 | Was the data analysis conducted with sufficient coverage of the identified sample? | X |  |  |  |
| 6 | Were valid methods used for the identification of the condition? | X |  |  |  |
| 7 | Was  the  condition  measured  in  a  standard,  reliable  way   for  all  participants? | X |  |  |  |
| 8 | Was there appropriate statistical analysis? | X |  |  |  |
| 9 | Was the response rate adequate, and if not, was the low response rate managed appropriately? | X |  |  |  |

**Overall appraisal: 88.9%, Include X Exclude □ Seek further info    □**

1. **Author: Moges et al Year: 2020 Record Number: 13**

- Reviewer 1: Yitayish Damtie(YD) Date: 6/10/2020

|  |  | Yes | No | Unclear | N/A |
| --- | --- | --- | --- | --- | --- |
| 1 | Was the sample frame appropriate to address the target population? |  |  | X |  |
| 2 | Were study participants sampled in an appropriate way? | X |  |  |  |
| 3 | Was the sample size adequate? | X |  |  |  |
| 4 | Were the study subjects and the setting described in detail? | X |  |  |  |
| 5 | Was the data analysis conducted with sufficient coverage of the identified sample? | X |  |  |  |
| 6 | Were valid methods used for the identification of the condition? | X |  |  |  |
| 7 | Was  the  condition  measured  in  a  standard,  reliable  way   for  all  participants? | X |  |  |  |
| 8 | Was there appropriate statistical analysis? | X |  |  |  |
| 9 | Was the response rate adequate, and if not, was the low response rate managed appropriately? | X |  |  |  |

- Reviewer 2: Bezawit Adane(BA) Date: 8/10/2020

|  |  | Yes | No | Unclear | N/A |
| --- | --- | --- | --- | --- | --- |
| 1 | Was the sample frame appropriate to address the target population? |  |  | X |  |
| 2 | Were study participants sampled in an appropriate way? | X |  |  |  |
| 3 | Was the sample size adequate? | X |  |  |  |
| 4 | Were the study subjects and the setting described in detail? | X |  |  |  |
| 5 | Was the data analysis conducted with sufficient coverage of the identified sample? | X |  |  |  |
| 6 | Were valid methods used for the identification of the condition? | X |  |  |  |
| 7 | Was  the  condition  measured  in  a  standard,  reliable  way   for  all  participants? | X |  |  |  |
| 8 | Was there appropriate statistical analysis? | X |  |  |  |
| 9 | Was the response rate adequate, and if not, was the low response rate managed appropriately? | X |  |  |  |

- Reviewer 3: Mastewal Arefaynie(MA) Date: 7/10/2020

|  |  | Yes | No | Unclear | N/A |
| --- | --- | --- | --- | --- | --- |
| 1 | Was the sample frame appropriate to address the target population? |  |  | X |  |
| 2 | Were study participants sampled in an appropriate way? | X |  |  |  |
| 3 | Was the sample size adequate? | X |  |  |  |
| 4 | Were the study subjects and the setting described in detail? | X |  |  |  |
| 5 | Was the data analysis conducted with sufficient coverage of the identified sample? | X |  |  |  |
| 6 | Were valid methods used for the identification of the condition? | X |  |  |  |
| 7 | Was  the  condition  measured  in  a  standard,  reliable  way   for  all  participants? | X |  |  |  |
| 8 | Was there appropriate statistical analysis? | X |  |  |  |
| 9 | Was the response rate adequate, and if not, was the low response rate managed appropriately? | X |  |  |  |

**Overall appraisal: 88.9%, Include X Exclude □ Seek further info    □**

1. **Author: Abebo et al Year: 2019 Record Number: 14**

- Reviewer 1: Yitayish Damtie(YD) Date: 6/10/2020

|  |  | Yes | No | Unclear | N/A |
| --- | --- | --- | --- | --- | --- |
| 1 | Was the sample frame appropriate to address the target population? |  |  | X |  |
| 2 | Were study participants sampled in an appropriate way? | X |  |  |  |
| 3 | Was the sample size adequate? | X |  |  |  |
| 4 | Were the study subjects and the setting described in detail? | X |  |  |  |
| 5 | Was the data analysis conducted with sufficient coverage of the identified sample? | X |  |  |  |
| 6 | Were valid methods used for the identification of the condition? | X |  |  |  |
| 7 | Was  the  condition  measured  in  a  standard,  reliable  way   for  all  participants? |  | X |  |  |
| 8 | Was there appropriate statistical analysis? | X |  |  |  |
| 9 | Was the response rate adequate, and if not, was the low response rate managed appropriately? | X |  |  |  |

- Reviewer 2: Bezawit Adane(BA) Date: 8/10/2020

|  |  | Yes | No | Unclear | N/A |
| --- | --- | --- | --- | --- | --- |
| 1 | Was the sample frame appropriate to address the target population? |  |  | X |  |
| 2 | Were study participants sampled in an appropriate way? | X |  |  |  |
| 3 | Was the sample size adequate? | X |  |  |  |
| 4 | Were the study subjects and the setting described in detail? | X |  |  |  |
| 5 | Was the data analysis conducted with sufficient coverage of the identified sample? | X |  |  |  |
| 6 | Were valid methods used for the identification of the condition? |  |  | X |  |
| 7 | Was  the  condition  measured  in  a  standard,  reliable  way   for  all  participants? | X |  |  |  |
| 8 | Was there appropriate statistical analysis? | X |  |  |  |
| 9 | Was the response rate adequate, and if not, was the low response rate managed appropriately? | X |  |  |  |

- Reviewer 3: Mastewal Arefaynie(MA) Date: 7/10/2020

|  |  | Yes | No | Unclear | N/A |
| --- | --- | --- | --- | --- | --- |
| 1 | Was the sample frame appropriate to address the target population? |  |  | X |  |
| 2 | Were study participants sampled in an appropriate way? | X |  |  |  |
| 3 | Was the sample size adequate? | X |  |  |  |
| 4 | Were the study subjects and the setting described in detail? | X |  |  |  |
| 5 | Was the data analysis conducted with sufficient coverage of the identified sample? | X |  |  |  |
| 6 | Were valid methods used for the identification of the condition? | X |  |  |  |
| 7 | Was  the  condition  measured  in  a  standard,  reliable  way   for  all  participants? | X |  |  |  |
| 8 | Was there appropriate statistical analysis? | X |  |  |  |
| 9 | Was the response rate adequate, and if not, was the low response rate managed appropriately? | X |  |  |  |

**Overall appraisal: 81.5%, Include X Exclude    □ Seek further info    □**
