# Supplementary material for: HIV risk behavior and associated factors among people living with HIV/AIDS in Ethiopia: A systematic review and meta-analysis
Source: PLoS One. 2022 Jul 28;17(7):e0269304. doi: 10.1371/journal.pone.0269304 (PMC9333449; doi:10.1371/journal.pone.0269304)
Supplement: Attachment — Submitted filename: Response to reviewer.docx [file pone.0269304.s005.docx]

**Point by point response for the rejected paper** PONE-D-20-39344

**Reviewer #1**

1. While the authors are clear that they are examining adult samples of people living with HIV the authors are not clear whether this includes same-sex transmission or same-sex sexual risk behaviors. Moreover, it is unclear what proportion of the composite sample are men, women or transgender. If the authors chose to focus on a specific gender or on exclusively different-sex sexual behaviors this should be explained and justified. If the authors included variation in these areas this should be noted and described.

***Response: Thank you for this important comment. We described whether our study focus on same-sex or different-sex transmission as well as the focus of our study with respect to the gender of study participants in line 117 -122 and table one of the revised manuscript.***

2. The authors examine the relationship between a few possible factors with sexual risk behavior (e.g. alcohol use, HIV status disclosure, stigma, etc.); however, in the background the authors do not establish the broader literature on these topics or set up why they are examining these relationships.

***Response: This is very nice observation. We justified the reason for examining the relationship between risky sexual behavior and alcohol intake, HIV status disclosure, and perceived stigma in the introduction section line 80-85 of the revised manuscript.***

Results:
3. While the authors indicate their broader definition of sexual risk, it isn’t clear in the results how these behaviors were measured differently across studies whether some of the studies used composite measures, or specific measures. Did the authors account for potential differences by measure? How did the authors approach harmonizing the measures for the purposes of meta-analysis? This would be useful information to include in Table 1. If there is gender variation or other unique characteristics of each sample it may also be useful to indicate this in the Table 1. I don’t have a sense of how heterogeneous or homogenous measurement was between these studies.

***Response: Many thanks for this key comment. We include all the concerns in Table 1 of our revised manuscript (Please see Table 1)***

4. It is unclear how alcohol consumption was measured in the studies that were used to assess the relationship between alcohol consumption and HIV risk behaviors.

***Response: We have tried to include how the included studies measured alcohol consumption under the outcome measurement section line 129 to 131 of the revised manuscript.***

Discussion:

5. Are there critiques or implications for how the relationships included in this analysis are being studied in Ethiopia? (Sampling, measurement, omissions, etc.).

***Response: Yes. We incorporated critiques in the discussion section line 271-273*.**

6. The authors briefly mention national program, as well as screening and treatment, but don’t clearly establish the current practices in Ethiopia in the discussion or how these results speak to those current practices. The implications could benefit from more discussion of this. Right now the authors have not emphasized the case for why this analysis is important and haven’t fully supported important implications being drawn from the analysis (beyond getting pooled estimates).

***Response: We have tried to address it in the discussion section line 291-298 and 304-308*.**

Figures and Diagrams:

7. The figures and diagrams appear to lack titles and are blurry such that it impacts the reader’s ability to interpret them. I recommend that the authors include titles and clearer images.

***Response: Thank you for your comment. According to PLOS ONE formatting style, figures are submitted as fig 1, fig 2… Without figure title and the respective figure titles are cited within the manuscript that is why the figures appear without titles. But we have tried our best to make the images clearer.***

General:

8. There are some typos and formatting issues throughout that may be remedied with a closer read.

***Response: The whole parts of the manuscript was revised for typing and formatting issues.***
