# Supplementary material for: HIV risk behavior and associated factors among people living with HIV/AIDS in Ethiopia: A systematic review and meta-analysis
Source: PLoS One. 2022 Jul 28;17(7):e0269304. doi: 10.1371/journal.pone.0269304 (PMC9333449; doi:10.1371/journal.pone.0269304)
Supplement: Attachment — Submitted filename: Response to Reviewers.docx [file pone.0269304.s006.docx]

**Douglas S. Krakower, MD**

**Academic Editor, PLOS ONE**

**RE: Manuscript ID:** PONE-D-21-17808 (HIV risk behavior and its association with alcohol intake, HIV status non-disclosure, and perceived stigma among people living with HIV/AIDS in Ethiopia: A systematic review and meta-analysis.)

**Dear Dr. Douglas S. Krakower,**

Thank you very much for your email and the comments/suggestions of the reviewers and academic editor. We have looked at the comments and have revised our paper accordingly. We hope our paper improved as a result of incorporating the reviewers' and academic editor's comments and suggestions.

Please find for your kind consideration the following:

- A rebuttal letter that responds to each point raised by the academic editor and reviewer labeled 'Response to Reviewers'. The point-by-point responses are written in *italic* font style.
- A revised manuscript with track changes labeled 'Revised Manuscript with Track Changes'.
- A revised paper without tracked changes labeled 'Manuscript'

While hoping that these changes would meet with your favourable consideration, we are happy to hear if there are more comments and suggestions. Please do not hesitate to let us know if you have any questions.

Yours Sincerely,

Metadel Adane

Department of Environmental Health, Wollo University

Dessie, Ethiopia

Tel:+251910336962

E-mail: [metadel.adane2@gmail.com](mailto:metadel.adane2@gmail.com)

**Point by point response**

**Editor Comments:**

I agree with the Reviewers' comments and addressing these will be a requirement for acceptance. In addition, the authors must revisit the concept of "risky" sexual behavior prior to acceptance. People living with HIV who use antiretroviral therapy and attain a suppressed viral load cannot transmit HIV through sexual contact, and this is a critical concept that is not highlighted in the article, and which must be integrated into any study on sexual behaviors among people living with HIV (or those who have sex with members of this population). Further, the term "risky" is stigmatizing, and so it is no longer appropriate for describing sexual behaviors. The term "HIV risk behaviors" is more acceptable. The authors have also not adequately discussed the reasons why people engage in what they define as "risky" behaviors; is it for sexual pleasure, because of alcohol use disorders, or other reasons that can help inform ways to improve HIV prevention intervention in Ethiopia? Finally, I agree that the services of a copy editing expert and more detailed proofreading will be essential to ensure that the paper is easy to read and suitable for publication in this journal.

***Response: Thank you for your constructive comment. We have tried to highlight this critical concept in the introduction section line 58-67. In addition, we have tried to use the term “HIV risk behavior” throughout the document. Furthermore, efforts were made to revise our document by language expert.***

**Reviewer #1:**

**1. Title**: change “adult HIV infected patients” to “adults living with HIV”

***Response: Thank you dear reviewer. We have amended it.***

**2. Key words**: revise to remove standard terms i.e. prevalence.

***Response: Thank you. We have removed it.***

**3. Terminology**: use correct terminology for “HIV infected patients”; use consistently: PLWHA

***Response: Thank you for your comment. We have tried to use the term “PLWHA” throughout our document accordingly.***

**Abstract**

4. Line 23: risky sexual practice does not increase HIV incidence; it increases the risk of HIV transmission.

***Response: Thank you very much for your constructive comments. We have tried to modify it accordingly.***

**Background**

5. Line 53: CDC only used once so no need for abbreviation.

***Response: Thank you for your suggestion. We have amended it.***

6. Line 54: lower case for “Sexually Transmitted Infection”. One does not contract AIDS but HIV, please edit the sentence.

***Response: Thank you. We have modified it accordingly.***

7. Line 60: Use abbreviation PLWHA

***Response: Thank you. The comment is accepted and addressed accordingly.***

8. Line 80-83: Consider revising or add to methods or discussion.

***Response: Thank you. We have revised it.***

**Methodology**

9. Line 118-124: What was the inclusion criteria? The section on inclusion criteria is not clear. Were studies included limited to those conducted in Ethiopia?

***Response: Many thanks for your comment. It is editorial problem. Studies included in this meta-analysis are limited to those conducted in Ethiopia. These are additional inclusion criteria’s.***

***Population****: This meta-analysis include studies conducted among male, female or both male and female adults living with HIV in Ethiopia.*

***Exposure****: PLWHA who took alcohol, disclosed their HIV sero-status and those who experienced perceived stigma.*

***Comparison****: PLWHA who didn’t take alcohol, patients with HIV sero-status non-disclosure and those without perceived stigma.*

***Outcome****: Studies assessed risky sexual practice as a primary outcome; whether it is same-sex or different-sex risk behavior.*

***Study design****: all types of observational studies (Cross-sectional, case-control, and cohort) were included.*

***Study setting****: all facility-based, as well as community-based studies*

***Time frame****: articles published from the beginning of 2000 up to September 30, 2020*

***Country****: studies conducted only in Ethiopia*

***Language****: articles written in the English language*

***Publication****: both published and unpublished articles were included in this study*.

10. Line 131-133: revise sentence e.g. Studies measured alcohol intake as “taking any alcoholic beverage in the last three months” [24], one study used “ever used alcohol” [35] and the remaining one study measured it as “alcohol consumption in the last 12 months” [27]

***Response: Thank you. We have tried to revise it accordingly.***

11. Line 135: Risky sexual practice?

***Response: Definitely, it is to mean risky sexual practice.***

12. Line 140: Six reviewers….

***Response: Thank you for your suggestion. We have corrected it.***

13. Line 141: reference the pretested data extraction format.

***Response: Thank you for your comment. But the comment is not clear for us. How can we reference it? If you want to see the format, the dataset is already uploaded as a supporting information so that you can see the data extraction form from the dataset. If this is not the case, make it clear for the next revision so that we will entertain it accordingly.***

14. Line 147-149: Were there any corresponding authors that were contacted for clarification?

***Response: Yes.***

15. Line 192-194: Were there any regional differences worth discussing?

***Response: Thank you for your valuable comment. Although it was not significant, the pooled prevalence of HIV risk behavior was relatively low among studies conducted in Addis Ababa as compared to studies conducted in SNNPR, Oromia and Amhara region.***

**Results**

16. Line 197: typo: Severe heterogeneity….

***Response: Thank you. We have amended it.***

17. Line 209-210: Revise the first two sentences. Was this meant to be a standalone paragraph?

***Response: Thank you for your suggestion. We have tried to revise it.***

18. Line 216-217: Please add a comment in your discussion regarding regions.

***Response: Thank you. We have tried to discuss the subgroup prevalence of HIV risk behavior across regions accordingly in line 235-237.***

19. Line 222: Use scientific writing… What does “a little bit high” mean? Please revise.

***Response: Thank you for your constructive comment. We have tried to revise it accordingly.***

20. Line 229: For OR, please use 1 decimal point throughout and be consistent.

***Response: Thank you. The comment is accepted and addressed accordingly.***

**Discussion**

21. Do not repeat your results in your discussion. You can still highlight specifics but limit repetition of results.

***Response: Thank you. We have amended it.***

22. Line 253-254: Is 39% prevalence high? Significant?

***Response: Yes. It is high as compared to different studies conducted before.***

**Reviewer #2:**

1. This a meta-analysis of the prevalence of sexual risk behavior among people living with HIV in Ethiopia. The researchers also examined the relationship between sexual risk behavior and alcohol use, stigma, and HIV disclosure. Although the study covers an important topic, it has some conceptual and methodological problems.

***Response: Thank you. We have tried to modify our document accordingly.***

2. The researchers justify the study by indicating that it will solve controversies regarding the factors that affect sexual risk behaviors in Ethiopia. However, there is no discussion of the differences the study intends to address. Moreover, although alcohol use and stigma may affect risk behaviors, moderators such as such as age, gender, access to treatment, and type of partner are likely to explain the heterogeneity in the reviewed studies. In fact, the characteristics and behaviors of the sample included in the review are not described.

***Response: Thank you for your important comment. We have tried to discuss the differences in the result of the primary studies regarding the factors that affect HIV risk behaviors in result section line 244-245, 253-254 and 262-263. Moreover, we have tried to conduct moderator analysis based on the sex of study participants. However, it is difficult to conduct moderator analysis based the patient age, access to treatment since all are on ART and type of sexual partner since the sexual partner is not specified on primary studies. However, the characteristics and behaviors of the samples included in the review are described on Table 1.***

3. The description of the outcome and predictor variables is incomplete. Risk behaviors include number of sexual partners, inconsistent condom use, and having had casual partners. How were these standardized? What happened with studies that included multiple risk-behavior measures or reported continuous outcomes (e.g., average number of sexual partners)? Given the high heterogeneity in the studies and the diverse indicators of risk behaviors, it would be important to conduct moderator analyses in terms of type of outcome measure, in addition to key demographic characteristics aside from the study setting.

***Response: Thank you for your important comment. We have tried to write the description of the outcome and predictor variables in method section line 135-152. In addition, we have conducted moderator analyses in terms of type of outcome measure and the sex of study participants as indicated in Table 3****.* ***But, we did not attempt to standardize definitions of number of sexual partners, inconsistent condom use, and having had casual partners rather we used them as used in each of the articles selected. None of the articles included in the review reported continuous outcomes.***

4. How were stigma and HIV status disclosure measured in the primary studies? Were the variables dichotomous?

***Response: Thank you for your constructive comment. Both stigma and HIV status disclosure were dichotomous variables. Primary studies measured HIV sero-status disclosure as “YES” if respondents told their HIV positive status to their recent sexual partner and “NO” if they didn’t. Primary studies calculated perceived stigma from respondents’ response to stigma-specific questions. It was measured as “having perceived stigma” if respondents scored greater than or equal to the mean value and “have no perceived stigma” if respondents scored below the mean value.***

5. The description of the quality assessment is incomplete, how many studies were excluded because of the quality assessment?

***Response: Thank you for your important comment. We have tried to revise the quality assessment section. In our study, all of the included articles scored 50% and more, thus all are included in the review.***

6. In the discussion, the extensive descriptions of the levels of risk behavior in different regions needs to be streamlined so it is easier to compare and interpret findings.

***Response: Thank you for your comment. We have tried to discuss the levels of risk behavior across regions in the discussion section line 286-292***

7. It is difficult to conclude that alcohol use affects sexual risk behaviors, given the measures that are included in the studies (e.g., lifetime alcohol use, alcohol use last 12 months).

***Response: Thank you for your constructive comment. It is the amount of alcohol used (rather than the timing of alcohol use) that largely determine the sexual behaviour of PLWHA. Since the amount of alcohol determine the HIV risk behaviour of PLWHA, It is also difficult to conclude that alcohol use in the last 3 months affects HIV risk behavior. In our opinion, it is PLWHA who have ever used alcohol and those used alcohol in the last 12 months who are likely to adapt and maintain the behaviour (alcohol use) and have a chance to be addicted with alcohol and hence practice sexual risk behaviour as compared to those who used alcohol recently. Due to these reason we can conclude that lifetime alcohol use, alcohol use last 12 months can affect sexual risk behaviors.***

8. I would recommend having the paper revised by a native English speaker or professional translator. There are lines that sound awkward or are unclear, and/or need to be revised in terms of word choice and punctuation. There is also some inconsistency in the use of terms. In addition, some paragraphs would benefit from reorganization.

***Response: The whole parts of the manuscript was revised by language expert for typing, formatting, word choice and punctuation issues. We also have tried to reorganize paragraphs and use terms consistently.***
