# Supplementary material for: HIV risk behavior and associated factors among people living with HIV/AIDS in Ethiopia: A systematic review and meta-analysis
Source: PLoS One. 2022 Jul 28;17(7):e0269304. doi: 10.1371/journal.pone.0269304 (PMC9333449; doi:10.1371/journal.pone.0269304)
Supplement: Attachment — Submitted filename: Response to Reveiwers.docx [file pone.0269304.s007.docx]

**Douglas S. Krakower, MD**

**Academic Editor, PLOS ONE**

**RE: Manuscript ID:** PONE-D-21-17808R1 (HIV risk behavior and its association with alcohol intake, HIV status non-disclosure, and perceived stigma among people living with HIV/AIDS in Ethiopia: A systematic review and meta-analysis.)

**Dear Dr. Douglas S. Krakower,**

Thank you very much for your email and the comments/suggestions of the reviewers and academic editor. We have looked at the comments and have revised our paper accordingly. We hope our paper improved as a result of incorporating the reviewers' and academic editor's comments and suggestions.

Please find for your kind consideration the following:

- A rebuttal letter that responds to each point raised by the academic editor and reviewer is labeled 'Response to Reviewers'. The point-by-point responses are written in *italic* font style.
- A revised manuscript with track changes labeled 'Revised Manuscript with Track Changes'.
- A revised paper without tracked changes labeled 'Manuscript'

While hoping that these changes would meet with your favorable consideration, we are happy to hear if there are more comments and suggestions. Please do not hesitate to let us know if you have any questions.

Yours Sincerely,

Metadel Adane

Department of Environmental Health, Wollo University

Dessie, Ethiopia

Tel:+251910336962

E-mail:[metadel.adane2@gmail.com](mailto:metadel.adane2@gmail.com)

**Point by point response**

**Editor’s comment**

**Comment 1**: Addressing the most recent reviewers' comments that relate to the statistical methods used and their interpretation and discussion, to ensure methodological rigor in the final published manuscript.

***Response: Thank you, dear editor. We have tried to address reviewers' comments accordingly.***

**Comment 2**: The term "HIV risk behavior" is currently used in an imprecise manner in the paper. Please be specific in the behaviors that you are describing. If you mean condom-less sex, sex with multiple partners, or some other behavior, please mention these in specific. "Unprotected" sex is not specific, as PLWH who have a suppressed viral load cannot transmit HIV to their sexual partners, so sex without condoms in this scenario is "protected" from HIV transmission (even if not from other STIs or pregnancy); please change to "condomless sex" if that is the intended meaning.

***Response: Thank you. Studies included in this meta-analysis did not address the same behavior. Some assessed risky sexual behavior, others assessed inconsistent condom use, condom unprotected sex, etc.….. So it is difficult to specify exactly those behaviors that are being addressed since the included studies assessed different behaviors. So the term that represents all these behaviors is risky sexual behavior. We used the term risky sexual behaviors to represent all these behaviors since risky sexual behavior according to the Center for Disease Control definition includes one or more of the following behaviors: sex without the use of condoms, inconsistent condom use, having multiple sexual partners, casual sex, sex with the influence of alcohol, and sexual exchange (exchange of money for sexual intercourse). Moreover, we have tried to use condomless sex to represent unprotected sex throughout the document.***

**Comment 3**: In line 60, the term "premise" is not appropriate, as this suggests that the assertion that follows is uncertain or untested. Please change to: "There is robust evidence that PLWH who are undetectable on ART cannot transmit HIV to their sexual partners."

***Response: Thank you, dear reviewer. We have amended it accordingly.***

**Comment 4**: Lines 61 to 67 suggest that the acquisition of drug-resistant HIV strains among PLWH is a major factor in the need for second-line ART. The totality of the literature does not support this assertion. Further, the references cited are generally more than a decade old, which ignores a much more robust set of studies from 2010 onward that do not support the authors' statements. Without a more evidence-based and updated argument, the introduction is not appropriate for publication. It would be appropriate to state that "However, PLWH may have challenges in accessing and adhering to ART, which can result in detectable viral loads and the potential for HIV transmission" with the support of new references.

***Response: Thank you, dear reviewer. The comment is accepted and addressed accordingly.***

**Comment 5**: Lines 80-82: The authors do not justify why the factors listed are the "most fundamental to intervene," and this strong assertion needs to be moderated. It would be more appropriate to state that these factors merit consideration as ones that could impact sexual behaviors and might be amenable to interventions.

***Response: Thank you for your constructive comment. We have amended it accordingly.***

**Comment 6**: Line 129: the authors need to justify why unpublished studies were included in the analyses.

***Response: Thank you dear reviewer for remembering an important issue. All the studies included in this meta-analysis were published articles. However, during the initial searching process, we found one unpublished study which has been posted in the research square. Due to this reason, we have said that “both published and unpublished studies were included in the review”. But finally, when we checked its publication status, it was already published in the journal. So in this study, only published studies were included. We have tried to revise the inclusion criteria in our revised manuscript.***

**Comment 7**: Lines 136-139: The authors need to state clearly how they operationalized alcohol use given the heterogeneous definitions used in prior studies. Because the definition for this and other covariates of interest, such as HIV status disclosure, were heterogeneous, this needs to be discussed as a major limitation when considering the study findings in the Discussion.

***Response: Thank you, dear reviewer. We have tried to revise the operational definition and discuss it as a limitation in the discussion section accordingly.***

**Comment 8**: In the Discussion, as above, the authors need to be far more precise when discussing HIV risk behaviors, and must specify exactly those behaviors that are being addressed, as opposed to the general term of "HIV risk behaviors," which can mean many different things. Unless all of the studies in lines 278-285 addressed the same behaviors, then it is not appropriate to make quantitative comparisons across studies as the authors have done.

***Response: Thank you for your constructive comment. As we have stated in the document, different literature defined risky sexual behavior as a behavior that includes one or more of the following behaviors: sex without the use of condoms, inconsistent condom use, having multiple sexual partners, casual sex, sex with the influence of alcohol, and sexual exchange (exchange of money for sexual intercourse). Studies included in this meta-analysis did not assess the same behaviors. Some assessed risky sexual behavior and others assessed inconsistent condom use or condom unprotected sex. So it is difficult to specify exactly those behaviors that are being addressed since they assessed different behaviors. Due to this reason, we used the term HIV risk behaviors to represent all these behaviors. We acknowledged it as a limitation in the revised manuscript.***

**Comment 9**: Line 39 and elsewhere: the term "safe sex" is outdated, non-specific, and not appropriate. Please remove this and specify exactly what is meant, such as how alcohol could influence decisions around condom use, status disclosure, or other factors that can affect HIV transmission.

***Response: Thank you, dear reviewer. We have amended it throughout the document.***

**Comment 10**: Line 347: The term "alcohol abuse" is stigmatizing; please change it to "alcohol use" or "alcohol use disorders" or similar. As above, please also remove "unsafe" as a term.

***Response: Thank you. The comment is accepted and addressed accordingly.***

**Comment 11**: The Discussion and Conclusion are missing a critical emphasis on the importance of promoting access and adherence to ART as a way to decrease HIV transmission, as suppressed viral loads are the most effective way to prevent transmission - even more than condoms or any social-behavioral interventions. The authors can discuss how counseling about alcohol, disclosure, and stigma is important to decrease HIV transmission among those without the use of ART, but ART needs to be mentioned front and center given the immense strength of evidence behind this biomedical strategy.

***Response: Thank you. We have tried to revise the discussion and conclusion section accordingly.***

**Reviewer #3**

**Comment 1**: I read with great interest the Manuscript titled "Risky sexual practice and its association with alcohol intake, HIV status disclosure, and perceived stigma among adult HIV infected patients in Ethiopia. A systematic review and meta-analysis" which falls within the aim of PLOSE ONE Journal. In my honest opinion, the topic is interesting enough to attract the readers' attention. The methodology is accurate and conclusions are supported by the data analysis. Nevertheless, the authors should clarify some points and improve the operational definition of some variables.

***Response: Thank you dear reviewer for your constructive comments. We have tried to revise the manuscript based on the comments of the reviewer accordingly.***

**Comment 2**: The rationale that the authors stated is not consistent across the document. For instance, in the abstract, the authors said “ this meta-analysis aimed to estimate the pooled prevalence of the risky sexual practice and its association with……" but in the background section page 4, lines 80-81 the authors said, " However, we only examine the association between risky sexual behavior and alcohol intake, HIV status disclosure, and perceived stigma since they were the most frequently mentioned factors and have controversial findings among the included studies”.

***Response: Thank you. We have amended it accordingly.***

**Comment 3**: How the authors operationalized alcohol consumption, perceived stigma, and HIV status disclosure is not clear. Their definition across the studies might be different and the authors should add one table concerning the definition of these variables in each study included in this review.

***Response: Thank you for your suggestion. The definition of perceived stigma and HIV status disclosure is similar across the included articles due for this reason, there is no need to put their definition in table 1. However, we have tried to put the definition of alcohol use across the studies in table 1 of our revised manuscript.***

**Comment 4:**In the abstract section, the authors said “In Ethiopia, findings regarding risky sexual practice have been inconsistent and inconclusive". However, they said nothing about this in the background section. Please, show us the inconsistency you found by elaborating on this section.

***Response: Thank you. We have tried to show the inconsistencies in the background section accordingly.***

**Reviewer #4:**

**Comment 1:** This is a good manuscript, clearly written, with the correct methodology and correct statistics for meta-analysis. However, it needs revision to be improved.

***Response: Thank you, dear reviewer. We have tried to revise our manuscript according to your comment.***

**Comment 2:** Tile: It was too long! "It’s association with alcohol intake, HIV status non-disclosure, and perceived stigma" This listed associated factor can be written concisely.

***Response: Thank you. We have modified the title to "HIV risk behavior and associated factors among people living with HIV/AIDS in Ethiopia: A systematic review and meta-analysis.”***

Abstract:

**Comment 3:**International databases such as Google Scholar, the Cochrane Library, HINARI, PubMed, CINAHL, and Global Health. Good to identify which one is a database and which one is a search engine? Furthermore, why are you using HINARI and Global Health? It is good to consider if you consider others, i.e., AJOL, WorldCat

***Response: Thank you, dear reviewer. Except for google scholar; Cochrane Library, HINARI, PubMed, CINAHL, and Global Health are databases. We used HINARI and Global Health since they are easily accessible (Wollo university has the password for the HINARI database due to this we can easily access other databases via HINARI). We have already missed AJOL and WorldCat in the current meta-analysis. We will consider them for our future experience.***

**Comment 4:**Why did you use the word "prevalence" for "HIV risk behavior"?

***Response: Thank you, dear reviewer. Some studies included in this study used the word "prevalence". So, we used this term in order not to miss those studies.***

Background

**Comment 5:** "There is also a premise that PLWHA who achieve and maintain viral load suppression cannot transmit HIV to their HIV-negative sexual partners [11]." I suggested instead of maintaining viral load suppression, changing to "undetectable viral load cannot pass HIV on through sex".

***Response: Thank you for your suggestion. We have amended it accordingly.***

**Comment 6:**There is previous SR and MA has already been performed (eg. Mekuriaw, B., Belayneh, Z., Molla, A. et al. A meta-analysis and systematic review20, 55 (in 2021).<https://doi.org/10.1186/s12954-021-00503-6>). What is the difference between this previous work (alcohol use and its determinants among adults living with HIV/AIDS) and the current work (alcohol use on HIV risk behavior)? Please provide information about the heterogeneity of this previous MA.

***Response: Thank you. There is a big difference between the previous work and the current one. The outcome variable for the previous work was alcohol use among PLWHA and the outcome variable for the current study is HIV risk behavior. Alcohol use was used as an independent predictor of HIV risk behavior in the current study as opposed to the previous work which was used as an outcome variable.***

**Comment 7:** More description of the "core problem" is required than justification.

***Response: Thank you. The comment is accepted and addressed accordingly.***

**Comment 8:** "Moreover, there is no single country-level figure estimating the pooled prevalence of HIV risk behavior among PLWHA in Ethiopia." Take a look at # 2 for an example.

***Response: Thank you for your suggestion. We have amended it accordingly.***

Methods:

**Comment 9:**Is there any amendments or changes? Did the authors write a protocol before doing this SR research (if any)? Additionally, there is a major difference in the description from the PROSPERO registration (CRD42020160018) e.g. The title at PROSPERO is "systematic review and meta-analysis on the magnitude and determinants of inconsistent condom use among adult HIV patients in Sub-Saharan Africa," which is very different from the title, which sets "HIV risk behavior and its association with alcohol intake, HIV status non-disclosure, and perceived stigma among people living with HIV/AIDS in Ethiopia: A systematic review and meta-analysis." Please provide an explanation and revision at PROSPERO.

***Response: Thank you for your constructive comment. Initially, we proposed to do SR research entitled "Systematic review and meta-analysis on the magnitude and determinants of inconsistent condom use among adult HIV patients in Sub-Saharan Africa," which was registered at PROSPERO with a registration number of CRD42020160018. However, due to the small number of articles, we have changed the title to "HIV risk behavior and its association with alcohol intake, HIV status non-disclosure, and perceived stigma among people living with HIV/AIDS in Ethiopia: A systematic review and meta-analysis.” We used the registration number of the former title to the current one without amending the protocol which may mislead the reader. We acknowledged the problem and have removed the registration status of the title from our manuscript.***

**Comment 10**: Search strategies: Why did the authors not use mesh terms in their search strategy? Moreover, the search strategies must be clearly stated in the annex (S2 File). You mention only PubMed, why not others? It should be clearly shown the search strategies by "PICO" or "PECO" or "CoCoP" and finally, use Boolean terms like "AND" or "OR." Then put in the exact date and time of the search with its findings. How do you get gray literature?

***Response: Thank you for your comment. We have amended the search strategy and tried to put the findings from each searching database in S2 File accordingly. Gray literature were identified by traditional Google search and Addis Ababa digital library search.***

**Comment 11**: Why did the authors collect studies only from 2000 to 2020? Shouldn't the result section have included data from 2008?

***Response: Thank you. We have tried to collect articles published from the beginning of 2000 to September 30, 2020, to get more articles assessing HIV risk behavior. However, during the searching processes, we found only articles published from 2008-to 2020.***

**Comment 12:**Authors should explain more data/information extraction selections in detail and place them in a table with the contents of the chosen article.

***Response: Thank you, dear reviewer. We have tried to include more data in the data extraction section accordingly. However, we haven’t seen the advantage of putting the data extraction section in tablet form since it is already attached as supporting information.***

**Comment 13:**More information about the quality assessment of selected articles is required.

***Response: Thank you. We have modified the quality assessment section according to your comment.***

**Comment 14:**Authors concluded heterogeneity in their MA by using the I2. The I2 is a value that quantifies heterogeneity, but the presence of significant heterogeneity should be done with the Q test and its respective p-value.

***Response: Thank you for your constructive comment. Although Q test is the usual way of assessing whether a set of single studies are homogeneous in the meta-analysis, it only informs us of the presence versus the absence of heterogeneity, but it does not report on the extent of such heterogeneity. In addition, Q has low power as a comprehensive test of heterogeneity especially when the number of studies is small (Gavaghan et al, 2000). Recently, the I^2^ index has been proposed to quantify the degree of heterogeneity in a meta-analysis. A study which is highlighted in yellow color (Huedo-Medina TB, Sánchez-Meca J, Marin-Martinez F, Botella J. Assessing heterogeneity in meta-analysis: Q statistic or I² index?. Psychological methods. 2006 Jun; 11(2):193) showed that the I^2^ index as a complement to the Q test due to this reason we can use I^2^ and its respective p-value to indicate the presence of significant heterogeneity***

**Comment 15:**The subgroup analysis, sensitivity analysis, and Meta-regression should be more explained in the data analysis method section as there is a finding based on this analysis in the result section.

***Response: Thank you, dear reviewer. We have explained them in the data analysis section accordingly.***

**Comment 16:**Authors are encouraged to test for group differences and within the group subgroups as well (a p-value for interaction between groups).

***Response: Thank you. Have tried to test for group differences and within the group subgroups among the included studies. In addition, we have to put a p-value for interaction between groups in table 2 of our revised manuscript.***

Results

**Comment 17:**The "systematic review" was really rushed. More explanation and description of SR is required.

***Response: Thank you. The comment is not clear for as. As much as possible, we have tried to put the detail explanation and description of each content of the systematic review in our manuscript.***

**Comment 18:** Please indicate the type of ES, e.g., figure 2.

***Response: Thank you. The comment is accepted and addressed accordingly in fig 2.***

Discussion

**Comment 19:** Please add more sentences on the implications of the main findings in this section.

***Response: Thank you. We have tried our best to add the implications of the main findings in the discussion section accordingly.***

**Comment 20:** Heterogeneity is very important across all analyses, even in the subgroup analyses. Authors are encouraged to discuss the manuscript's limit.

***Response: Thank you. We have tried to discuss it as a limitation in the discussion section.***

**Comment 21:**Is there any strength in the manuscript? Please explain this, e.g., adding a body of knowledge, and a methodological perspective.

***Response: Thank you. We have tried to put the strength of our study in the discussion section.***
